# COVID-19 pneumonia on chest X-rays: Performance of a deep learning-based computer-aided detection system

**DOI:** 10.1371/journal.pone.0252440

**Published:** 2021-06-07

**Authors:** Eui Jin Hwang, Ki Beom Kim, Jin Young Kim, Jae-Kwang Lim, Ju Gang Nam, Hyewon Choi, Hyungjin Kim, Soon Ho Yoon, Jin Mo Goo, Chang Min Park

**Affiliations:** 1 Department of Radiology, Seoul National University College of Medicine, Seoul, Korea; 2 Department of Radiology, Seoul National University Hospital, Seoul, Korea; 3 Department of Radiology, Daegu Fatima Hospital, Daegu, Korea; 4 Department of Radiology, Keimyung University School of Medicine, Daegu, Korea; 5 Department of Radiology, School of Medicine, Kyungpook National University, Daegu, Korea; 6 Institute of Radiation Medicine, Seoul National University Medical Research Center, Seoul, Korea; Korea National University of Transportation, REPUBLIC OF KOREA

## Abstract

Chest X-rays (CXRs) can help triage for Coronavirus disease (COVID-19) patients in resource-constrained environments, and a computer-aided detection system (CAD) that can identify pneumonia on CXR may help the triage of patients in those environment where expert radiologists are not available. However, the performance of existing CAD for identifying COVID-19 and associated pneumonia on CXRs has been scarcely investigated. In this study, CXRs of patients with and without COVID-19 confirmed by reverse transcriptase polymerase chain reaction (RT-PCR) were retrospectively collected from four and one institution, respectively, and a commercialized, regulatory-approved CAD that can identify various abnormalities including pneumonia was used to analyze each CXR. Performance of the CAD was evaluated using area under the receiver operating characteristic curves (AUCs), with reference standards of the RT-PCR results and the presence of findings of pneumonia on chest CTs obtained within 24 hours from the CXR. For comparison, 5 thoracic radiologists and 5 non-radiologist physicians independently interpreted the CXRs. Afterward, they re-interpreted the CXRs with corresponding CAD results. The performance of CAD (AUCs, 0.714 and 0.790 against RT-PCR and chest CT, respectively hereinafter) were similar with those of thoracic radiologists (AUCs, 0.701 and 0.784), and higher than those of non-radiologist physicians (AUCs, 0.584 and 0.650). Non-radiologist physicians showed significantly improved performance when assisted with the CAD (AUCs, 0.584 to 0.664 and 0.650 to 0.738). In addition, inter-reader agreement among physicians was also improved in the CAD-assisted interpretation (Fleiss’ kappa coefficient, 0.209 to 0.322). In conclusion, radiologist-level performance of the CAD in identifying COVID-19 and associated pneumonia on CXR and enhanced performance of non-radiologist physicians with the CAD assistance suggest that the CAD can support physicians in interpreting CXRs and helping image-based triage of COVID-19 patients in resource-constrained environment.

## Introduction

The Coronavirus disease (COVID-19) pandemic has posed a major threat to health care systems worldwide. As of April, 2021, COVID-19 has affected more than 126 million people worldwide, causing more than 2.8 million deaths [1]. Although COVID-19 patient may show various symptoms including fever, cough, and dyspnea [2], more than half of infected patients can be asymptomatic [3], which may lead to delayed diagnosis and important cause of disease transmission [4]. Therefore, early identification of infected patients and adequate isolation is extremely important to interrupt human-to-human transmission and prevent morbidity and mortality caused by COVID-19 [5].

The primary diagnostic test for COVID-19 is a reverse transcriptase-polymerase chain reaction (RT-PCR) on nasopharyngeal swabs for virus detection [6, 7]. Radiologic examinations including chest X-ray (CXR) and chest CT may show findings of pneumonia, including consolidations and ground-glass opacities [8–10], however, since considerable proportion of patients may show normal radiologic finding (up to 40% in CXR) especially in early phase of the disease, CXRs and chest CTs are currently not recommended for screening or diagnosis of COVID-19 [11–14]. However, the massive COVID-19 outbreak has resulted in a shortage of medical resources [15–17], limiting timely testing of RT-PCR. Recent international consensus statements suggested that radiologic examinations can be used as a triage tool in resource-constrained environments [11, 12].

CXR has been the primary radiologic examination for pneumonia. Especially for highly contagious diseases such as COVID-19, CXR has an advantage over chest CT in preventing transmission, since transportation of patients can be minimized with portable radiography units, and disinfection of the scanners and environment is relatively easy [11, 13]. However, CXRs have low sensitivity and high inter-reader variability for COVID-19 pneumonia compared to chest CTs [8, 18–20]. In a previous study, CXRs showed a median sensitivity of 25% for opacities identified on chest CTs, and the sensitivity ranged from 20% to 50% across radiologists [19].

Recent studies have reported that artificial intelligence systems using deep learning techniques can detect various diseases on CXRs, showing comparable performance to expert radiologists [21–24]. These computer-aided detection systems (CADs) may help imaging-based triage of patients in resource-constrained environments where expert radiologists are not available, and improve accuracy and inter-reader variability of physicians’ CXR interpretations [25, 26]. Several recent studies have demonstrated that deep learning-based CAD can identify COVID-19 on CXRs with radiologist-level performance [27, 28] and can be implemented for clinical practice [29]. However, the performance of a commercialized, clinically-available CAD in identifying COVID-19 and associated pneumonia has been rarely investigated and whether the CAD can enhance the performance of physicians and their inter-reader agreement

Therefore, in this study, we evaluated the performance of a commercialized, clinically-available CAD in identifying COVID-19 and associated pneumonia. We compared the performance of the CAD with physicians’ interpretation, and investigated whether the CAD can enhance the performance of physicians’ interpretation and their inter-reader agreement.

## Materials and methods

The present study was approved by the institutional review boards of all participating institutions, with a waiver of patients’ informed consent.

### Patients

**Fig 1:**
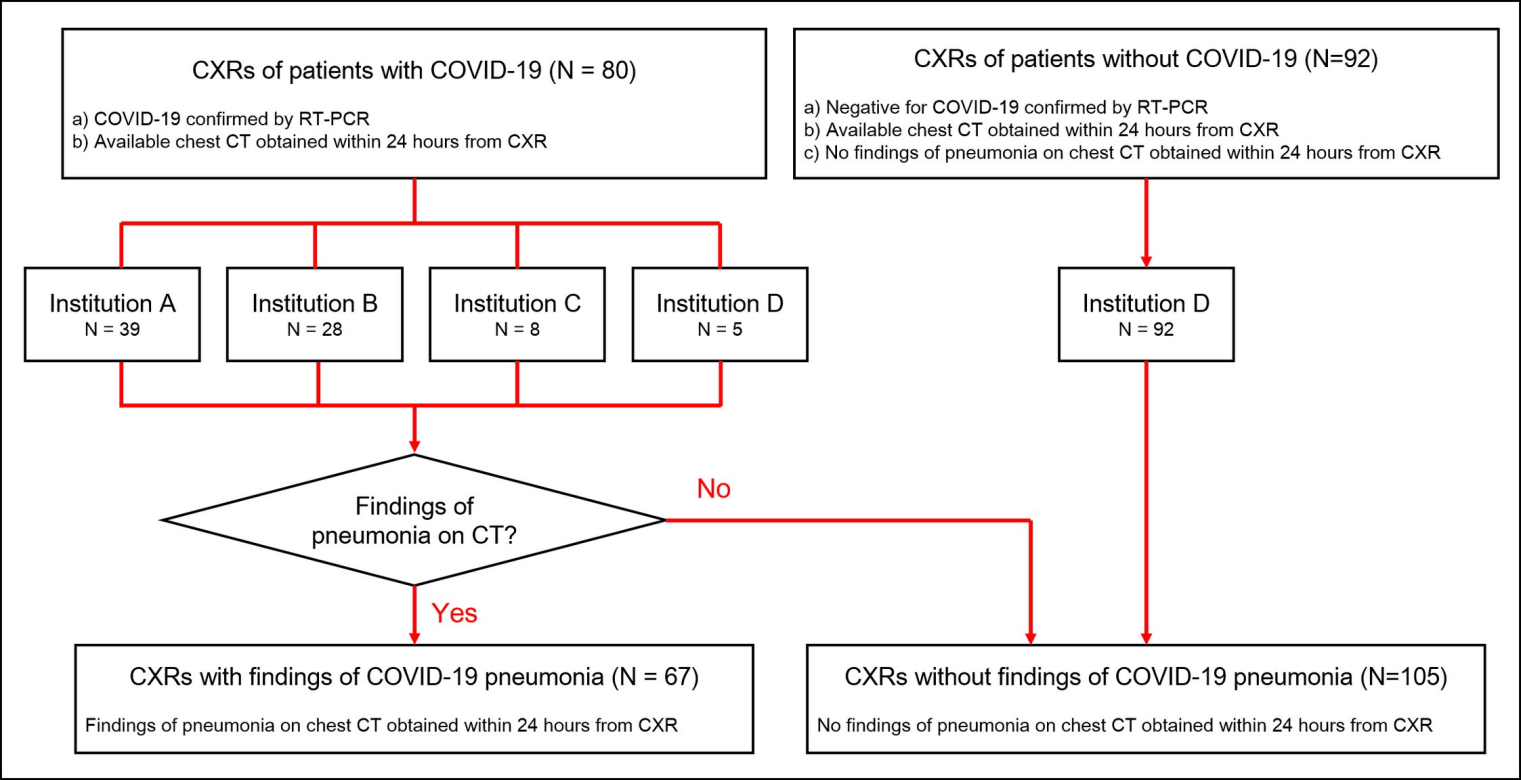
Flow diagram for patients’ inclusion. A total of 80 patients with COVID-19 were retrospectively included from four institutions, while 92 patients without COVID-19 were included from single institution. Among patients with COVID-19, 67 exhibited findings of pneumonia on chest CT obtained within 24 hours from CXR, while the other 13 patients did not exhibited any findings of pneumonia on chest CT.

Patients with COVID-19 were retrospectively included from four institutions (Daegu Fatima Hospital, Daegu, Korea; Keimyung University Dongsan Medical Center, Daegu, Korea; Kyungpook National University Hospital, Daegu, Korea; and Seoul National University Hospital, Seoul, Korea) with following inclusion criteria: a) COVID-19 patients confirmed by RT-PCR between January 20th and March 20th, 2020; and b) patients underwent CXR and chest CT within 24 hours (Fig 1).

Patients without COVID-19 were also retrospectively included from a single institution (Seoul National University Hospital) with following inclusion criteria: a) patients with negative RT-PCR result for COVID-19; b) patients underwent CXR and chest CT within 24 hours; c) patients without any abnormality suggesting pneumonia on chest CT (Fig 1).

### Deep learning-based CAD

We used a commercialized deep learning-based CAD (Lunit INSIGHT CXR 2, Lunit Inc., Seoul, Korea) for evaluating the CXRs. The CAD was designed to detect pulmonary nodules or masses, pulmonary infiltrates, and pneumothoraxes on CXRs. The CAD was initially trained using 54,221 normal CXRs and 35,613 abnormal CXRs (including 6,903 CXRs with pneumonia) [21], and was not specifically trained with CXRs from COVID-19 patients.

**Fig 2:**
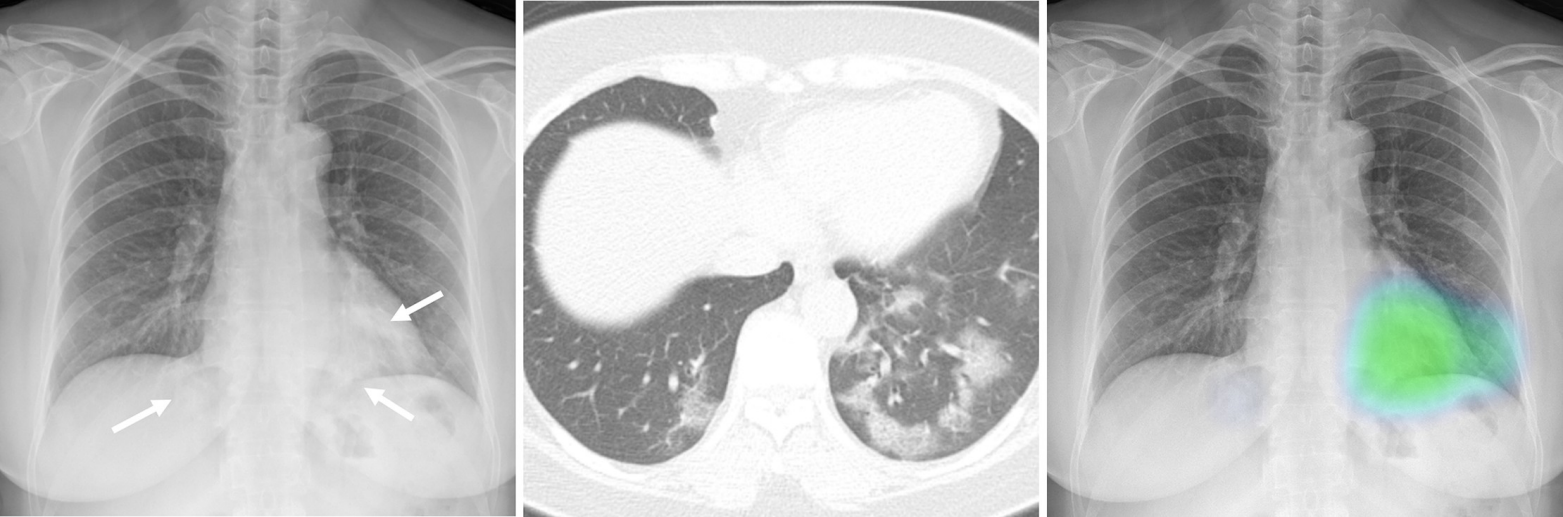
Representative case with COVID-19 pneumonia. A CXR (**A**) of a patient with confirmed COVID-19 shows patchy infiltrates in both lower lung fields (arrows). The corresponding chest CT image (**B**) obtained in the same day with the CXR shows multifocal patchy ground-glass opacities in both lower lobes of the lung. The CT severity score of the patient was 13. The CAD system correctly detected pulmonary infiltrates with a probability score of 56% (**C**). In the reader-alone interpretation, four thoracic radiologists correctly identified the abnormality while none of the non-radiologist physicians identified the abnormality. In the CAD-assisted interpretation, all five thoracic radiologists and four non-radiologist physicians identified the abnormality.

**Fig 3:**
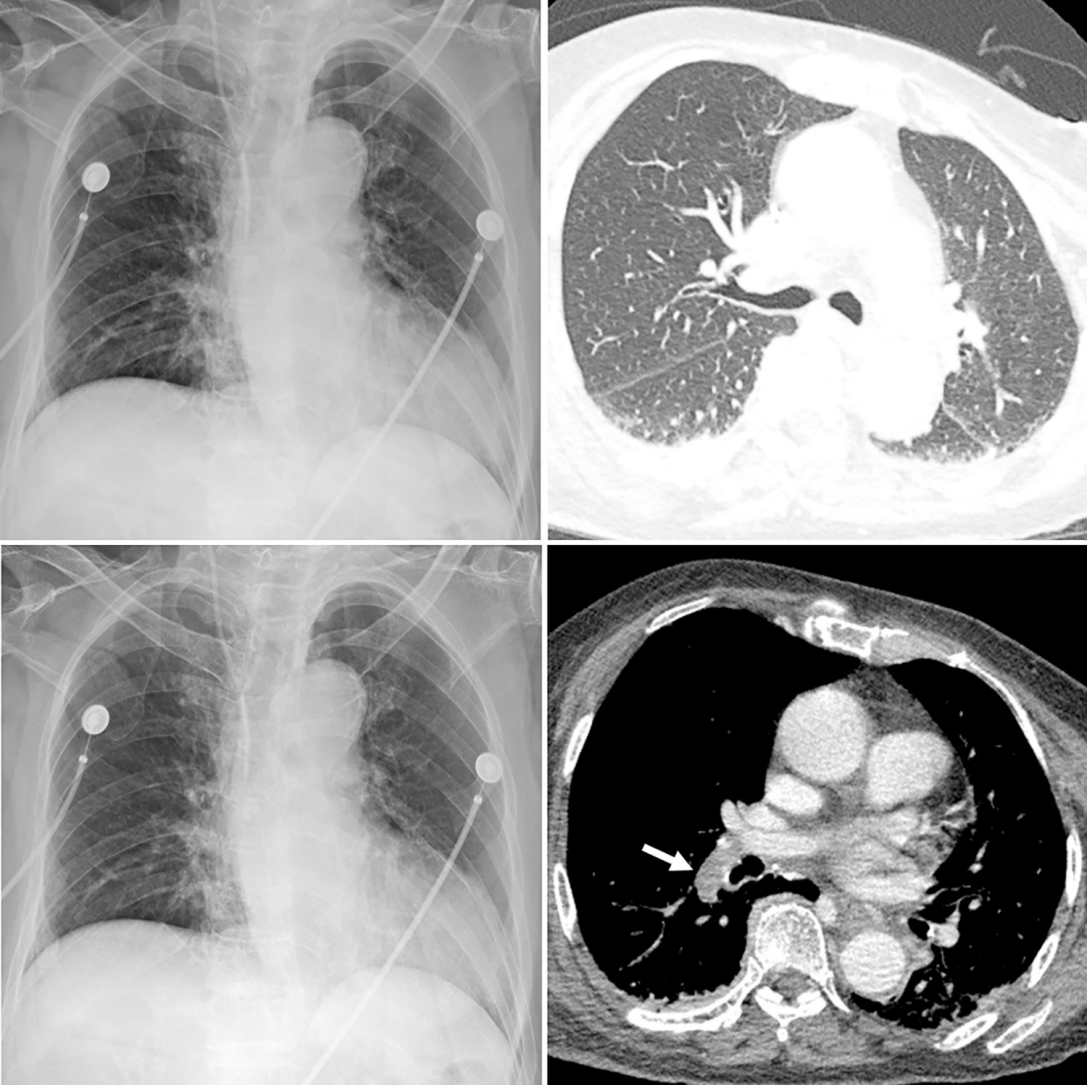
Representative case without COVID-19 pneumonia. A CXR (**A**) and corresponding chest CT (**B**) of a patient with fever and dyspnea but negative RT-PCR result for COVID-19 show no pulmonary abnormality suggestive of pneumonia. The CAD system did not detect any abnormalities in the CXR and the probability score was 13% (**C**). In the reader-alone interpretation, four thoracic radiologists and four non-radiologist physicians misclassified the CXR as having findings of pneumonia. In the CAD-assisted interpretation, only one thoracic radiologist and two non-radiologist physicians made false-positive classification of the CXR. Mediastinal window CT image (**D**) show pulmonary embolism in the right descending pulmonary artery (arrow), presumed cause of patients’ symptom.

The CAD provided a probability score between 0 and 100% for the presence of abnormality on each CXR, with a heat map overlaid on the CXR for the localization of abnormality when the probability score was 15% or greater (Figs 2 and 3).

### Definition of reference standards

For evaluation of CXR interpretation, we defined two different classes of reference standard: a) the diagnosis of COVID-19 by RT-PCR; and b) the presence of pneumonia on chest CT.

To define the reference standard for the presence of pneumonia, two thoracic radiologists (E.J.H and C.M.P, 9 and 21 years of experience in CXR and chest CT interpretation respectively) who were blinded to the CAD results reviewed the chest CT images, which were obtained within 24 hours from the CXRs. The two radiologists determined whether there was any finding of pneumonia on CT, in consensus.

Features of pneumonia on chest CT (the presence of consolidations, ground-glass opacities, and pleural effusion, and the bilaterality of pneumonia) were evaluated by one thoracic radiologist (E.J.H). For evaluation of the extent of pneumonia, a previously reported scoring system [30] based on the segmental involvement of the infiltrates (CT severity score, range 0–40) was adopted. For evaluation of CT severity score, bilateral lungs were divided into 20 regions based on lobar and segmental anatomy. Opacities of all 20 lung regions were visually evaluated by the thoracic radiologist on CT, and score 0, 1, and 2 were assigned if parenchymal opacities involved 0%, <50%, and ≥50% of each region, respectively. Finally, the CT severity score was defined as the sum of the individual scores of the 20 lung regions [30].

### Reader test

A total of 10 readers (5 thoracic radiologists [5–29 years of experience in CXR interpretation] and 5 non-radiologist physicians) participated in a reader test. All physicians independently interpreted the CXRs, to address whether there was any abnormality suggestive of pneumonia. Physicians were informed to provide a five-point scale score for each CXR regarding the presence and absence of the abnormality suggestive of pneumonia: a) score 1, definitely absent; b) score 2, probably absent; c) score 3, equivocal; d) score 4, probably present; and e) score 5, definitely present. All readers were informed that CXRs were obtained from patients suspected for COVID-19, however, the readers were blinded to other clinical information.

At first, each physician interpreted the CXRs at their discretion (reader-alone interpretation). Subsequently, physicians were provided with the CAD results and asked to modify their original decision as needed (CAD-assisted interpretation).

### Subgroup analyses

To evaluate performances of the CAD and readers for detection of pneumonia in patients with different clinical and radiologic findings, we compared sensitivities of the CAD and readers for identification of findings of pneumonia, in following subgroups: a) patients with symptom duration >5 days versus ≤5 days; b) patients with versus without consolidation on their CTs; and c) patients with CT severity score >10 versus ≤10.

### Statistical analyses

We evaluated the performances of positive interpretation results by the CAD and physicians for the prediction of positive reference standards: The diagnosis of COVID-19 by RT-PCR, and the presence of pneumonia on CT. Area under the receiver operating characteristic curves (AUCs), sensitivities, and specificities were used for performance evaluations. For evaluating sensitivity and specificity, the CAD results with a probability score ≥15% were considered as positive results, while physicians’ scores ≥3 were considered as positive interpretations. Average AUCs of multiple readers were obtained and compared using multiple reader multiple cases receiver operating characteristic analyses, as suggested by Obuchowski and Rockette [31]. Average sensitivities and specificities of multiple readers were estimated using generalized estimating equations. Inter-reader agreements among physicians in five-point scale scores and in binary classifications were evaluated with Fleiss’ kappa coefficient.

All statistical analyses were done with R (version 3.6.3, R project for statistical computing, Vienna, Austria). A *P*-value <0.05 was considered to indicate a statistically significant difference.

## Results

### Demographic and radiologic information

A total of 172 CXRs from 172 patients (87 men; median age, 66 years [interquartile range (IQR), 57–75 years]) were included in the study. Among them, 80 (46.5%) were COVID-19 patients confirmed by RT-PCR. 94.7% (163/172) of patients had symptoms suggestive of acute respiratory illness, and the median time interval between symptom onset and CXR acquisition was 3 days (IQR, 1–6 days) (Table 1).

**Table 1:** Clinical and radiological information of patients.

	Patients with positive RT-PCR (n = 80)	Patients with negative RT-PCR (n = 92)	*P*-values	Patients with pneumonia on chest CTs (n = 67)	Patients without pneumonia on chest CTs (n = 105)	*P*-values
Proportion of male patients ^a^	50.0% (40/80)	51.1% (47/92)	.887	49.3% (33/67)	51.4% (54/105)	.781
Age (years)^b^	64 (55–71)	68 (60–77)	.025	64 (57–73)	66 (56–76)	.402
Proportion of symptomatic patients ^a^	96.3% (77/80)	92.4% (85/92)	.342	97.0% (65/67)	92.4% (97/105)	.319
Duration since symptom onset (days)^b^^,^ ^c^	5 (3–7)	1 (0–2)	< .001	5 (3–7)	1 (0–3)	< .001
Findings of pneumonia on chest CTs^a^	83.8% (67/80)	0% (0/92)	< .001	100% (67/67)	0% (0/105)	< .001

Abbreviations: CT, computed tomography; RT-PCR, Reverse transcriptase-polymerase chain reaction.

^a^ Numbers in the parentheses indicate numerators/denominators.

^b^ Data indicate medians (interquartile ranges).

^c^ Three asymptomatic patients were excluded.

Based on chest CTs, 83.8% (67/80) of COVID-19 patients had findings of pneumonia, while the other 16.2% (13/80) of COVID-19 patients and all the 92 non-COVID-19 patients did not exhibit findings of pneumonia. Among 67 patients with pneumonia on CTs, ground-glass opacities, consolidations, and pleural effusion were observed in 100% (67/67), 47.8% (32/67), and 6.0% (4/67) of patients. 85.1% (57/67) of patients showed bilateral involvement. The median CT severity score of patients with pneumonia was 13 (IQR, 7–21).

### CAD versus reader-alone interpretations

**Fig 4:**
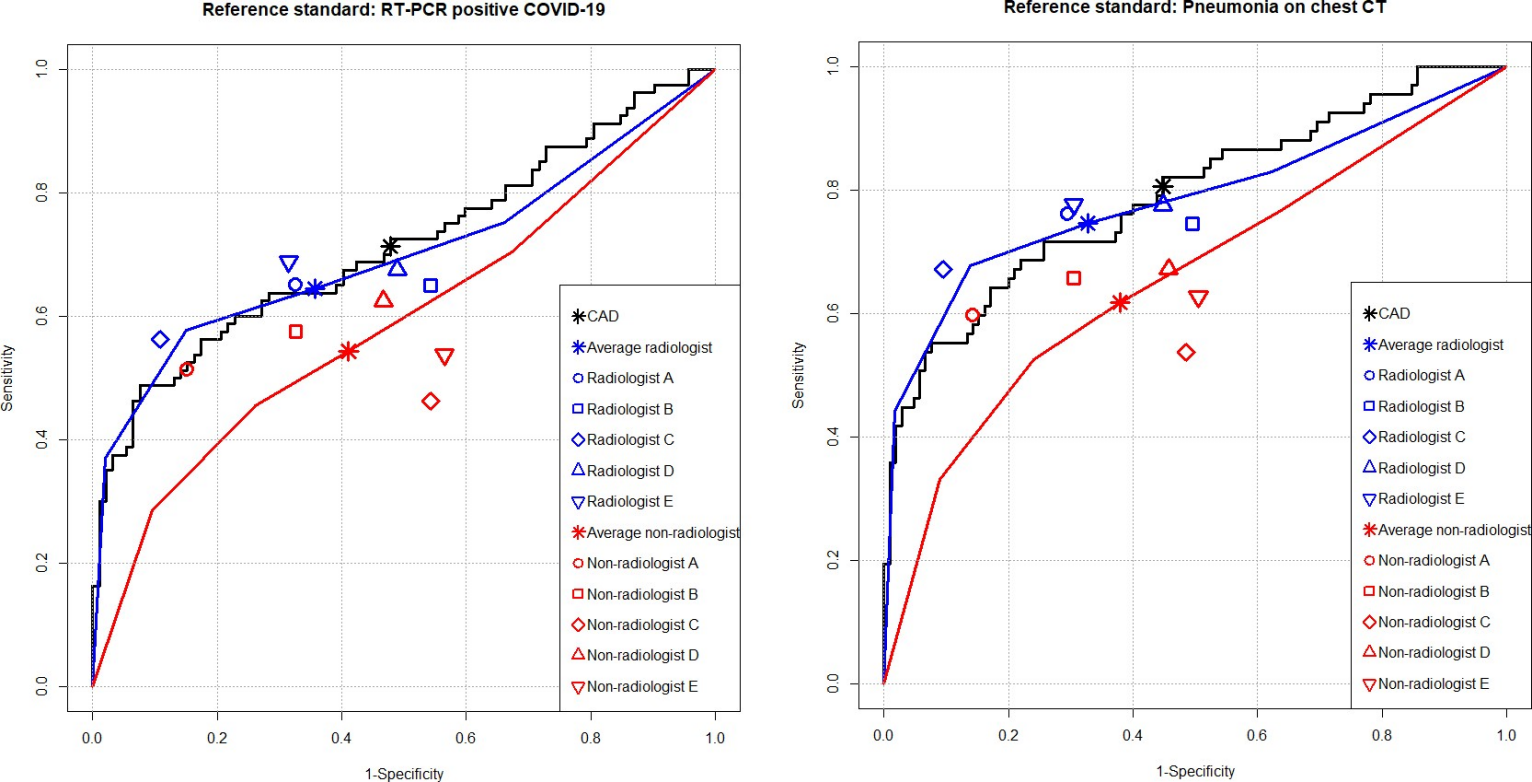
Performance of the CAD versus reader-alone interpretations. For identification of RT-PCR-positive COVID-19 patients (**A**), the CAD exhibited AUC of 0.714 (black line), which did not significantly differ from that of thoracic radiologists (0.701, blue line) but significantly higher than that of non-radiologist physicians (0.584, red line). For identification of pneumonia defined on chest CT (**B**), the CAD exhibited AUC of 0.790 (black line), which was not significantly different from that of thoracic radiologists (0.784, blue line), but significantly higher than that of non-radiologist physicians (0.650, red line).

For identification of RT-PCR-positive COVID-19 patients, the CAD exhibited an AUC, sensitivity, and specificity of 0.714 (95% confidence interval [CI], 0.641–0.781), 71.3% (95% CI, 61.3–81.2%), and 52.2% (95% CI, 42.0–62.4%), respectively. The AUC of the CAD did not significantly differ from that of average thoracic radiologist (0.701 [95% CI, 0.619–0.783]; *P* = .712), while significantly higher than that of average non-radiologist physician (0.584 [95% CI, 0.469–0.699]; *P* = .031). The sensitivity of the CAD was not significantly different form that of average thoracic radiologist (64.5% [95% CI, 54.7–73.2%]; *P* = .228), while significantly higher than that of average non-radiologist physician (54.3% [95% CI, 44.9–63.4%]; *P* = .003). The specificity of the CAD was significantly lower than that of average thoracic radiologist (64.3% [95% CI, 59.9–68.6]; *P* = .032), and did not significantly differ from that of average non-radiologist physician (58.9% [95% CI, 54.4–63.3%]; *P* = .236). Regarding individual readers, the CAD exhibited significantly higher AUC than four non-radiologist physicians, significantly higher sensitivity than one thoracic radiologist and four non-radiologist physicians. However, the specificity of the CAD was significantly lower than those of three thoracic radiologists and two non-radiologist physicians (Table 2, Fig 4).

**Table 2:** The CAD and physician’s performances with reference standard of diagnosis of COVID-19 by RT-PCR.

	Reader-alone interpretation ^a^						CAD-assisted interpretation ^b^					
	AUC	*P*-value	Sensitivity	*P*-value	Specificity	*P*-value	AUC	*P*-value	Sensitivity	*P*-value	Specificity	*P*-value
CAD	0.714	Ref.	71.3%	Ref.	52.2%	Ref.	NA	NA	NA	NA	NA	NA
	(0.641–0.781)		(61.3–81.2%)		(42.0–62.4%)							
Average thoracic radiologist	0.701	.712	64.5%	.228	64.3%	.032	0.699	.815	63.0%	.659	69.3%	.107
	(0.619–0.783)		(54.7–73.2%)		(59.9–68.6%)		(0.621–0.777)		(53.2–71.9%)		(65.0–73.4%)	
Thoracic radiologist A	0.675	.245	65.0%	.166	67.4%	.008	0.678	.890	58.8%	.096	75.0%	.035
	(0.600–0.745)		(54.5–75.5%)		(57.8–77.0%)		(0.602–0.747)		(48.0–69.5%)		(66.2–83.8%)	
Thoracic radiologist B	0.640	.044	65.0%	.166	45.7%	.317	0.655	.153	65.0%	>.999	56.5%	.012
	(0.564–0.712)		(54.5–75.5%)		(35.5–55.8%)		(0.579–0.726)		(54.5–75.5%)		(46.4–66.7%)	
Thoracic radiologist C	0.739	.433	56.3%	.003	89.1%	< .001	0.733	.581	55.0%	.317	88.0%	.705
	(0.667–0.803)		(45.4–67.1%)		(45.4–67.1%)		(0.660–0.797)		(44.1–65.9%)		(81.4–94.7%)	
Thoracic radiologist D	0.688	.452	67.5%	.439	51.1%	.827	0.693	.801	68.8%	.655	60.9%	.013
	(0.613–0.756)		(57.2–77.8%)		(40.9–61.3%)		(0.618–0.761)		(58.6–78.9%)		(50.9–70.8%)	
Thoracic radiologist E	0.763	.156	68.8%	.564	68.5%	.014	0.736	.204	67.5%	.655	66.3%	.683
	(0.693–0.825)		(58.6–78.9%)		(59.0–78.0%)		(0.664–0.800)		(57.2–77.8%)		(56.6–76.0%)	
Average non-radiologist physician	0.584	.031	54.3%	.003	58.9%	.236	0.664	.006	61.8%	.031	65.2%	.048
	(0.469–0.699)		(44.9–63.4%)		(54.4–63.3%)		(0.580–0.748)		(52.3–70.4%)		(60.8–69.4%)	
Non-radiologist physician A	0.688	.478	51.3%	< .001	84.8%	< .001	0.745	.008	60.0%	.008	83.7%	.655
	(0.613–0.756)		(40.3–62.2%)		(77.4–92.1%)		(0.673–0.809)		(49.3–70.7%)		(76.1–91.2%)	
Non-radiologist physician B	0.639	.025	57.5%	.008	67.4%	.011	0.667	.214	60.0%	.480	69.6%	.564
	(0.562–0.710)		(46.7–68.3%)		(57.8–77.0%)		(0.591–0.737)		(49.3–70.7%)		(60.2–79.0%)	
Non-radiologist physician C	0.559	.040	46.3%	< .001	45.7%	.289	0.619	.465	57.5%	.002	64.1%	.001
	(0.481–0.634)		(35.3–57.2%)		(35.5–55.8%)		(0.542–0.692)		(46.7–68.3%)		(54.3–73.9%)	
Non-radiologist physician D	0.592	.025	62.5%	.172	53.3%	853	0.671	.005	71.3%	.108	51.1%	.655
	(0.515–0.667)		(51.9–73.1%)		(43.1–63.5%)		(0.595–0.740)		(61.3–81.2%)		(40.9–61.3%)	
Non-radiologist physician E	0.558	< .001	53.8%	.002	43.5%	.182	0.618	.029	60.0%	.132	57.6%	.016
	(0.480–0.633)		(42.8–64.7%)		(33.3–53.6%)		(0.541–0.691)		(49.3–70.7%)		(47.5–67.7%)	

Abbreviations: AUC, area under receiver operating characteristic curve; CAD, computer-aided detection system; NA, not applicable.

Numbers in the parentheses indicate 95% confidence intervals

^a^ *P*-values indicate comparison with the CAD

^b^ *P*-values indicate comparison with the reader-alone interpretations

For identification of pneumonia with the reference standard of CT, the CAD exhibited an AUC, sensitivity, and specificity of 0.790 (95% CI, 0.722–0.849), 80.6% (95% CI, 71.1–90.0%), and 55.2% (95% CI, 45.7–64.7%), respectively. The AUC of the CAD did not significantly differ from that of average thoracic radiologist (0.784 [95% CI, 0.713–0.856]; *P* = .839), while significantly higher than that of average non-radiologist physician (0.650 [95% CI, 0.544–0.756]; *P* = .016). The sensitivity of the CAD was not significantly different from that of average thoracic radiologist (74.6% [95% CI, 69.7–79.0%]; *P* = .268), while significantly higher than that of average non-radiologist physician (61.8% [95% CI, 52.5–66.8%]; *P* = .001). The specificity of the CAD was significantly lower than that of average thoracic radiologist (67.2% [95% CI, 63.1–71.1]; *P* = .023), and did not significantly differ from that of average non-radiologist physician (62.1% [95% CI, 57.9–66.2%]; *P* = .195). Regarding individual readers, the CAD exhibited significantly higher AUC than four non-radiologist physicians, significantly higher sensitivity than one thoracic radiologist and five non-radiologist physicians. However, the CAD exhibited significantly lower specificity than three thoracic radiologists and two non-radiologist physicians (Table 3, Fig 4).

**Table 3:** The CAD and physician’s performances with reference standard of pneumonia on chest CT.

	Reader-alone interpretation ^a^						CAD-assisted interpretation ^b^					
	AUC	*P*-value	Sensitivity	*P*-value	Specificity	*P*-value	AUC	*P*-value	Sensitivity	*P*-value	Specificity	*P*-value
CAD	0.790	Ref.	80.6%	Ref.	55.2%	Ref.	NA	NA	NA	NA	NA	NA
	(0.722–0.849)		(71.1–90.0%)		(45.7–64.7%)							
Average thoracic radiologist	0.784	.839	74.6%	.268	67.2%	.023	0.789	.524	74.0%	.860	72.4%	.069
	(0.713–0.856)		(69.7–79.0%)		(63.1–71.1%)		(0.721–0.858)		(69.1–78.4%)		(68.4–76.0%)	
Thoracic radiologist A	0.778	.691	76.1%	.317	70.5%	.005	0.777	.918	70.1%	.157	78.1%	.021
	(0.709–0.838)		(65.9–86.3%)		(61.8–79.2%)		(0.709–0.838)		(59.2–81.1%)		(70.2–86.0%)	
Thoracic radiologist B	0.738	.137	74.6%	.206	50.5%	.423.	0.759	.025	76.1%	.317	61.0%	.008
	(0.666–0.802)		(64.2–85.0%)		(40.9–60.0%)		(0.688–0.821)		(65.9–86.3%)		(51.6–70.3%)	
Thoracic radiologist C	0.799	.772	57.2%	.012	90.5%	< .001	0.802	.759	65.7%	.317	89.5%	.705
	(0.731–0.856)		(55.9–78.4%)		(84.9–96.1%)		(0.734–0.859)		(54.3–77.0%)		(83.7–95.4%)	
Thoracic radiologist D	0.773	.609	77.6%	.564	55.2%	>.999	0.785	.581	79.1%	.655	63.8%	.013
	(0.703–0.833)		(67.6–87.6%)		(45.7–64.7%)		(0.716–0.844)		(69.4–88.8%)		(54.6–73.0%)	
Thoracic radiologist E	0.833	.156	77.6%	.480	69.5%	.019	0.824	.660	79.1%	.564	69.5%	>.999
	(0.769–0.886)		(67.6–87.6%)		(60.7–78.3%)		(0.759–0.878)		(69.4–88.8%)		(60.7–78.3%)	
Average non-radiologist physician	0.650	.016	61.8%	.001	62.1%	.195	0.738	< .001	71.0%	.011	67.8%	.048
	(0.544–0.756)		(52.5–66.8%)		(57.9–66.2%)		(0.660–0.815)		(57.0–75.7%)		(63.7–71.7%)	
Non-radiologist physician A	0.750	.251	59.7%	< .001	85.7%	< .001	0.809	.011	70.1%	.008	84.8%	.655
	(0.678–0.812)		(48.0–71.4%)		(79.0–92.4%)		(0.742–0.865)		(59.2–81.1%)		(77.9–91.6%)	
Non-radiologist physician B	0.697	.037	65.7%	.008	69.5%	.009	0.739	.071	70.1%	.257	72.4%	.405
	(0.622–0.765)		(54.3–77.0%)		(60.7–78.3%)		(0.667–0.803)		(59.2–81.1%)		(59.2–81.1%)	
Non-radiologist physician C	0.517	< .001	53.7%	< .001	51.4%	.493	0.699	< .001	67.2%	.003	67.6%	.001
	(0.439–0.593)		(41.8–65.7%)		(41.9–61.0%)		(0.625–0.767)		(55.9–78.4%)		(58.7–76.6%)	
Non-radiologist physician D	0.635	< .001	67.2%	.020	54.3%	.866	0.739	< .001	79.1%	.046	53.3%	.835
	(0.558–0.707)		(55.9–78.4%)		(44.8–63.8%)		(0.666–0.803)		(69.4–88.8%)		(43.8–62.9%)	
Non-radiologist physician E	0.652	< .001	62.7%	.005	49.5%	.330	0.703	.074	68.7%	.206	61.0%	.028
	(0.576–0.723)		(51.1–74.3%)		(40.0–59.1%)		(0.628–0.770)		(57.5–79.8%)		(51.6–70.3%)	

Abbreviations: AUC, area under receiver operating characteristic curve; CAD, computer-aided detection system; NA, not applicable; Ref., reference.

Numbers in the parentheses indicate 95% confidence intervals

^a^ *P*-values indicate comparison with the CAD

^b^ *P*-values indicate comparison with the reader-alone interpretations

### Reader-alone interpretations versus CAD-assisted interpretations

**Fig 5:**
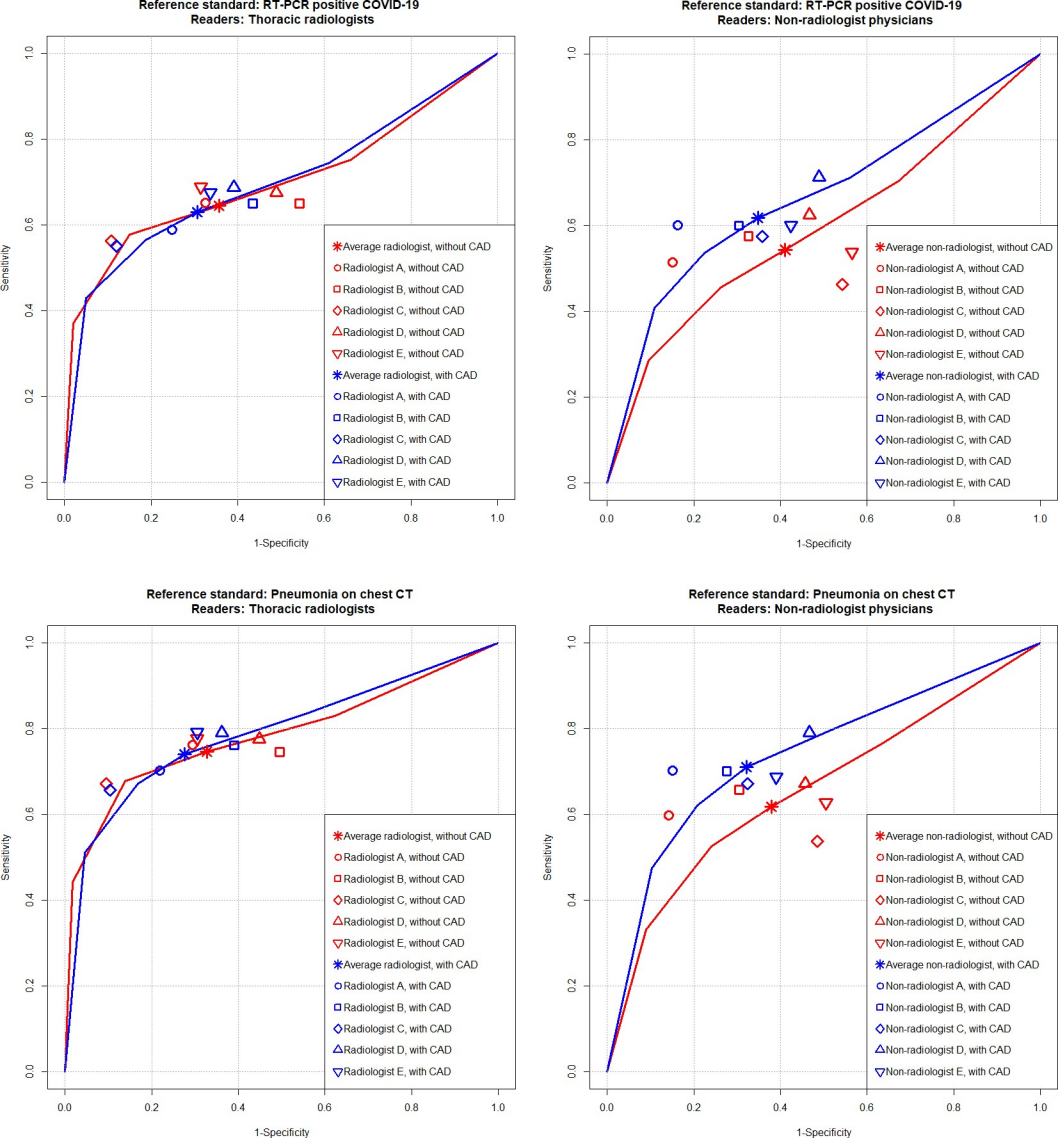
Performance of physician alone versus CAD-assisted interpretations. For identification of RT-PCR positive COVID-19 patients, the AUCs of thoracic radiologists did not significantly differ between reader-alone (red line) and CAD-assisted interpretations (blue line) (0.701 vs. 0.699; *P* = .815) (**A**), while the AUC non-radiologist physicians was significantly improved in the CAD-assisted interpretation (blue line) compared to the reader-alone interpretation (red line) (0.584 vs. 0.664; *P* = .006) (**B**). For identification of pneumonia defined on chest CT, the AUCs of thoracic radiologists also did not significantly differ between reader-alone (red line) and CAD-assisted interpretations (blue line) (0.784 vs. 0.789; *P* = .524) (**C**), while the AUC non-radiologist physicians was significantly improved in the CAD-assisted interpretation (blue line) compared to the reader-alone interpretation (red line) (0.650 vs. 0.738; *P* = .003) (**D**).

In the CAD-assisted interpretation, AUC (0.699 [95% CI, 0.621–0.777]; *P* = .815), sensitivity (63.0% [95% CI, 53.2–71.9%]; *P* = .659), and specificity (69.3% [95% CI, 65.0–73.4%]; *P* = .107) of average thoracic radiologist did not significantly differ from those in the reader-alone interpretation, for identification of COVID-19 patients with the reference standard of RT-PCR result. Meanwhile, all the AUC (0.664 [95% CI, 0.580–0.748]; *P* = .006), sensitivity (61.8% [95% CI, 52.3–70.4%]; *P* = .031), and specificity (65.2% [95% CI, 60.8–69.4%]; *P* = .048) of average non-radiologist physician were significantly improved in the CAD-assisted interpretation, with reference standard of RT-PCR result. Regarding individual readers, significant improvement of readers’ AUCs, sensitivities, and specificities were observed in three (three non-radiologist physicians), two (two non-radiologist physicians), and five (three thoracic radiologists and two non-radiologist physicians) readers, respectively in the CAD-assisted interpretation (Table 2, Fig 5)

As for the identification of pneumonia with the reference standard of CT, AUC (0.789 [95% CI, 0.721–0.858]; *P* = .524), sensitivity (74.0% [95% CI, 69.1–78.4%]; *P* = .860), and specificity (72.4% [95% CI, 68.4–76.0%]; *P* = .069) of average thoracic radiologist in the CAD-assisted interpretation did not significantly differ from those in the reader-alone interpretation. Meanwhile, AUC (AUC (0.738 [95% CI, 0.660–0.815]; *P* = .003), sensitivity (71.0% [95% CI, 57.0–75.7%]; *P* = .011), and specificity (67.8% [95% CI, 63.7–71.7%]; *P* = .048) of average non-radiologist physicians exhibited significant improvement in the CAD-assisted interpretation. Regarding individual readers, significant improvement of readers’ AUCs, sensitivities, and specificities were observed in four (one thoracic radiologist and three non-radiologist physicians), three (three non-radiologist physicians), and five (three thoracic radiologists and two non-radiologist physicians) readers, respectively in the CAD-assisted interpretation (Table 3, Fig 5).

As for inter-reader agreement, the CAD-assisted interpretation exhibited better agreement (Fleiss’ kappa coefficient, 0.322 [95% CI, 0.320–0.344] for the five-point-scale scores, and 0.688 [95% CI, 0.665–0.710] for the binary classification) compared to the reader-alone interpretations (Fleiss’ kappa coefficient, 0.209 [95% CI, 0.197–0.220] for the five-point-scale scores, and 0.510 [95% CI, 0.488–0.533] for the binary classification), without overlapping 95% CIs.

### Sensitivities in different subgroups

In the reader-alone interpretations, the readers exhibited significantly higher sensitivities for patients with a symptom duration >5 days (77.7%), for patients with consolidations on their CTs (73.4%), and for patients with CT severity scores >10 (84.5%), compared to those whose symptom duration was ≤5 days (59.4%; *P* < .001), those who did not have consolidations on their CTs (63.4%; *P* = .005), and those with CT severity scores ≤10 (46.9%; *P* < .001), respectively (Table 4).

**Table 4:** Sensitivities varied by clinical and radiological findings.

	CAD	*P*-values	Reader-alone interpretation*	*P*-values
Patients with a duration since symptom onset ≤5 days (n = 34)	71.4% (53.7–85.4%)	Ref.	59.4% (54.1–64.5)	Ref.
Patients with a duration since symptom onset >5 days (n = 31)	90.3% (74.2–98.0%)	.068^†^	77.7% (72.8–82.0%)	< .001^†^
Patients with consolidation on their chest CTs (n = 32)	90.6% (75.0–98.0%)	Ref.	73.4% (68.3–78.0%)	Ref.
Patients without consolidation on their chest CTs (n = 35)	71.4% (53.7–85.4%)	.065^‡^	63.4% (58.3–68.3%)	.005^‡^
Patients with a CT severity score ≤10 (n = 29)	69.0% (49.2–84.7%)	Ref.	46.9% (41.2–52.7%)	Ref.
Patients with a CT severity score >10 (n = 38)	89.5% (75.2–97.1%)	.059^§^	84.5% (80.5–87.8%)	< .001^‡^

Abbreviations: CAD, computer-aided detection system; CT, computed tomography; Ref., reference

*Data are pooled results of all 10 readers.

^†^Comparison with patients with a symptom duration >5 days

^‡^Comparison with patients without consolidation on their chest CTs

^§^Comparison with patients with a CT severity score >10

The CAD exhibited similar trends with physicians, and exhibited a higher sensitivity for patients with a symptom duration >5 days (90.3%), for patients with consolidations on their CTs (90.6%), and for patients with CT severity scores >10 (89.5%), compared to those whose symptom duration was ≤5 days (71.4%; *P* = .068), those who did not have consolidations on their CTs (71.4%; *P* = .065), and those with CT severity scores ≤10 (69.0%; *P* = .059), although the differences did not reach statistical significance.

## Discussion

Herein, we evaluated the performance of a commercialized deep learning-based CAD for identification of CXRs from RT-PCR positive COVID-19 patients and those with associated pneumonia proven by chest CT and compare it with those of thoracic radiologists and non-radiologist physicians. The performance of CAD (AUC, 0.714 and 0.790 for RT-PCR positive COVID-19 and associated pneumonia, respectively) was similar with those of thoracic radiologists (AUC, 0.712 and 0.784 for RT-PCR positive COVID-19 and associated pneumonia, respectively), and higher than those of non-radiologist physicians (AUC, 0.584 and 0.650 for RT-PCR positive COVID-19 and associated pneumonia, respectively).

Since CXRs and chest CTs of COVID-19 patients may appear normal, especially in the early stages of the disease [9, 32, 33], diagnosing COVID-19 using CXRs or CTs may be inappropriate [11–14]. In our study population, 16.3% of COVID-19 patients did not exhibit any findings of pneumonia on CTs. Not surprisingly, the performances of the CAD and readers against RT-PCR results were unsatisfactory. Sensitivities of the CAD (71.3%) and thoracic radiologists (56.3–68.8%) were comparable to previously reported sensitivity (69%) of baseline CXRs by Wong et al [18].

In spite of limited diagnostic performance compared to RT-PCR testing, identification of radiologic findings of pneumonia is still clinically important for the following reasons: first, in situations with limited medical resources due to the outbreak, a timely diagnosis with an RT-PCR test can be limited. Since the results of radiologic examinations can be obtained faster than RT-PCR results, it can aid timely clinical decision-making in resource-constrained environments. Second, radiological findings of pneumonia may precede positive RT-PCR results [18, 34–36]. In situations where there is a high pre-test probability, identification of pneumonia via radiologic examinations can help early diagnosis and allow for isolation in order to prevent further transmission. Third, since the extent of radiological findings of pneumonia can mirror the clinical severity of COVID-19 [30, 37, 38], radiological findings of pneumonia may aid hospitalization and intensive care decision-making, and may be utilized for monitoring the severity of the disease. The radiologist-level performance of the CAD for identification of findings of pneumonia suggests that it may facilitate the triage of CXRs with COVID-pneumonia, especially when interpretations from expert radiologists are limited or unavailable.

The CAD evaluated in the present study was not specifically trained for findings of COVID-19. Instead, it was trained for various types of abnormalities including pulmonary nodules and infiltrates. The reasonably high performance of the CAD indicates that an existing versatile CAD can be utilized for the detection of COVID-19 pneumonia. Indeed, radiographic findings of COVID-19 pneumonia include bilateral ground-glass opacities and consolidation [8–10, 39], which has substantial overlap with pneumonia from other etiologies. Although it is difficult to directly compare the performance across different studies because of difference in test datasets, recent studies where deep learning-based CADs that were specifically trained for COVID-19 pneumonia reported higher AUCs compared to our results (AUCs, 0.81–0.99, Table 5) for the identification of CXRs from COVID-19 patients [27, 28, 40, 41]. Additional training of the CAD with COVID-19 CXRs may improve the performance.

**Table 5:** Comparison of performance of CAD for identifying COVID-19 with results from different studies.

	Present study	Murphy et al. [27]	Wehbe et al. [28]	Castiglioni et al. [40]	Minaee et al. [41]
Specific training for COVID-19	No	Yes	Yes	Yes	Yes
Number of test dataset	172	454	2214	110	3100
Prevalence of COVID-19	46.5%	49.1%	53.9%	67.3%	3.2%
AUC	0.71	0.81	0.90	0.81	0.99
Sensitivity	71%	60%	75%	80%	98%
Specificity	52%	85%	93%	81%	93%

Abbreviations: CAD, computer-aided detection system; COVID-19, Coronavirus disease

In addition to stand-alone performance, we also observed that the CAD may enhance the performances of readers. Although thoracic radiologists did not exhibit significant improvement in performance in the CAD-assisted interpretation, less-experienced non-radiologist physicians exhibited significantly improved AUCs, sensitivities, and specificities in the CAD-assisted interpretations. Our results suggest that the CAD may help less-experienced readers to identify subtle findings of pneumonia on CXRs. In addition, the substantial improvement of inter-reader agreement using the CAD suggests that CAD can also help reduce variability among physicians in terms of CXR interpretations.

Regarding sensitivities in different subgroups, the CAD exhibited similar trends to physicians. Higher sensitivity in patients with consolidations and higher CT severity scores may be related to better visibility of pulmonary infiltrates on CXRs. A previous study reported that the entire burden of pneumonia reflecting both the extent and density of pneumonia on CTs was a significant factor for the visibility of pneumonia on CXRs [19]. Higher sensitivities in patients with longer time intervals since their symptom onset (>5days) may also be associated with the extent of pneumonia. Previous studies reported that the extent of pneumonia on CXR tended to increase until 2 weeks after symptom onset [30, 39]. Considering that imaging-based triage is indicated in patients with moderate to severe clinical symptoms [11], and disease extents in CTs reflect the clinical severity of diseases [30], we believe the CAD can help triage COVID-19 patients with CXRs.

Limitations exist in the present study. Despite being a multi-center study, our study included a limited number of patients. In addition, we included patients with available chest CT, as a reference standard for findings of pneumonia. Therefore, patients in our study did not fully reflect actual clinical situation, and the generalizability of our result may be limited. Additionally, the reader tests performed in our study also did not fully reflect actual clinical practice, where radiologists and clinicians may interpret CXRs in different environments from the reader test and can use the additional clinical and laboratory information. Therefore, the performances of the physicians in our study may not be fully reproducible in actual practice.

## Conclusion

A commercialized, clinically-available deep learning-based CAD exhibited similar performances CAD (AUC, 0.714 and 0.790 for RT-PCR positive COVID-19 and associated pneumonia, respectively) with thoracic radiologists (AUC, 0.712 and 0.784 for RT-PCR positive COVID-19 and associated pneumonia, respectively) in identifying COVID-19 and associated pneumonia on CXRs, and outperformed non-radiologist physicians (AUC, 0.584 and 0.650 for RT-PCR positive COVID-19 and associated pneumonia, respectively). It also enhanced the detection performances of non-radiologist physicians (AUC, 0.584 to 0.664 and 0.650 to 0.738 for RT-PCR positive COVID-19 and associated pneumonia, respectively) and inter-reader agreement (Fleiss’ kappa coefficient, 0.510 to 0.688). We believe that the CAD system can help less-experienced physicians to identify findings of pneumonia associated with COVID-19 on CXRs and to reduce variability among physicians in CXR interpretations. The CAD may facilitate imaging-based triage of COVID-19 patients in resource-constrained environment.

## Supporting information

S1 File Data. (XLSX)
